# Supplementary material for: Positive allosteric modulators of lecithin: Cholesterol acyltransferase adjust the orientation of the membrane-binding domain and alter its spatial free energy profile
Source: PLoS Comput Biol. 2021 Mar 15;17(3):e1008426. doi: 10.1371/journal.pcbi.1008426 (PMC7993845; doi:10.1371/journal.pcbi.1008426)
Supplement: S1 Text — Supporting Tables and Figures Table A The average number of hydrophobic interactions between drugs and nearby amino acids as mean (SD). Table B Accessible surface areas (ASA) of drugs in relevant contexts as mean (SD). Fig A The free-energy profiles (top) and umbrella sampling converge (bottom) for compounds 6 and 8 calculated by utilizing the umbrella sampling technique. Fig B The umbrella sampling histogram overlaps for compound 6 (10–50 ns) and 8 (50–90 ns). Fig C Steered molecular dynamics analysis with the force constant of 500 kJ/mol*nm showing the distance of the α-carbon atom of MET66 from the initial position as a function of time Fig D The converge of AWH simulations analyzed using simulation intervals of 100 ns. (DOCX) [file pcbi.1008426.s001.docx]

**SUPPLEMENTARY INFORMATION**

**Positive allosteric modulators of lecithin:cholesterol acyltransferase adjust the orientation of the membrane-binding domain and alter its spatial free energy profile**

Akseli Niemelä and Artturi Koivuniemi*

Division of Pharmaceutical Biosciences, Faculty of Pharmacy, University of Helsinki, Helsinki, Finland

***Correspondence**

Artturi Koivuniemi

The Division of Pharmaceutical Biosciences, Faculty of Pharmacy, University of Helsinki, Helsinki, Finland

Tel: +358451217612

E-mail: [artturi.koivuniemi@helsinki.fi](mailto:artturi.koivuniemi@helsinki.fi)

**SUPPLEMENTARY INFORMATION**

**Table A** The average number of hydrophobic interactions between drugs and nearby amino acids as mean (SD).

**Table B** Accessible surface areas (ASA) of drugs in relevant contexts as mean (SD).

**Fig A** The free-energy profiles (top) and umbrella sampling converge (bottom) for compounds 6 and 8 calculated by utilizing the umbrella sampling technique.

**Fig B** The umbrella sampling histogram overlaps for compound 6 (10-50 ns) and 8 (50-90 ns).

**Fig C** Steered molecular dynamics analysis with the force constant of 500 kJ/mol*nm showing the distance of the α-carbon atom of MET66 from the initial position as a function of time

**Fig D** The converge of AWH simulations analyzed using simulation intervals of 100 ns.

**Table A**

|  | Drug-1B | Drug-2A | Drug-2B | Drug-3 | Drug-6 | Drug-8 | Drug-9 |
| --- | --- | --- | --- | --- | --- | --- | --- |
| LEU40 | 0 (0) | 0.061 (0.252) | 0.005 (0.07) | 0 (0) | 0.001 (0.032) | 0.004 (0.064) | 0.03 (0.176) |
| MET49 | 3.641 (2.611) | 2.134 (1.861) | 2.255 (1.861) | 2.479 (1.992) | 0.592 (0.792) | 1.817 (1.725) | 2.327 (1.943) |
| CYS50 | 0 (0) | 0.082 (0.33) | 0 (0) | 1.147 (0.48) | 1.103 (0.564) | 1.067 (0.467) | 0.001 (0.032) |
| TYR51 | 4.63 (2.851) | 6.759 (3.041) | 4.591 (2.974) | 7.388 (3.456) | 6.509 (2.872) | 7.156 (3.24) | 6.485 (3.225) |
| PHE58 | 1.969 (1.378) | 1.998 (1.505) | 1.718 (1.443) | 1.492 (1.377) | 2.163 (1.337) | 1.636 (1.491) | 1.667 (1.549) |
| ILE60 | 0.03 (0.217) | 0.135 (0.568) | 0.141 (0.522) | 0.055 (0.298) | 0.043 (0.267) | 0.148 (0.521) | 0.14 (0.512) |
| ASP63 | 1.412 (0.896) | 1.082 (0.892) | 1.151 (0.874) | 1.331 (0.913) | 1.28 (1.28) | 0.089 (0.383) | 1.157 (0.878) |
| ASN65 | 3.876 (2.237) | 2.385 (1.575) | 2.022 (1.615) | 2.802 (1.897) | 4.226 (2.156) | 4.13 (1.621) | 2.006 (1.235) |
| MET66 | 1.226 (1.287) | 1.779 (1.295) | 1.976 (1.324) | 1.533 (1.211) | 1.193 (1.193) | 2.778 (1.358) | 2.046 (1.324) |
| LEU68 | 0.679 (1.231) | 2.36 (1.724) | 1.794 (1.656) | 1.814 (1.633) | 0.255 (0.578) | 2.402 (1.745) | 1.809 (1.649) |
| PRO69 | 0.752 (1.538) | 1.259 (1.562) | 1.026 (1.264) | 0.947 (1.452) | 0 (0) | 0.713 (1.313) | 1.497 (1.516) |
| LEU70 | 0.194 (0.727) | 0.009 (0.115) | 0.004 (0.077) | 0.031 (0.225) | 0.012 (0.126) | 0.206 (0.535) | 0.013 (0.139) |
| GLY71 | 2.461 (1.052) | 2.04 (1.291) | 2.31 (1.105) | 2.226 (1.209) | 2.275 (0.99) | 2.928 (1.041) | 2.068 (1.16) |
| CYS74 | 2.959 (1.123) | 2.374 (1.432) | 2.825 (1.044) | 3.236 (1.297) | 3.029 (1.075) | 3.086 (1.15) | 3.457 (1.424) |
| TRP75 | 3.885 (1.107) | 4.281 (1.233) | 4.056 (1.207) | 3.922 (1.208) | 3.77 (1.075) | 1.778 (1.545) | 3.774 (1.217) |
| ASN78 | 0 (0) | 0.026 (0.171) | 0 (0) | 0.002 (0.045) | 0 (0) | 0.009 (0.115) | 0.009 (0.115) |

**Table B**

|  | ASA of drug | Solvent ASA of drug in MBD | ASA of drug binding cleft |
| --- | --- | --- | --- |
| Drug-1B | 6.475 (0.185) | 1.748 (0.259) | 4.727 (0.319) |
| Drug-2A | 6.209 (0.181) | 1.276 (0.242) | 4.934 (0.302) |
| Drug-2B | 6.158 (0.186) | 1.405 (0.211) | 4.753 (0.281) |
| Drug-3 | 6.162 (0.186) | 1.444 (0.25) | 4.717 (0.312) |
| Drug-6 | 4.583 (0.154) | 0.581 (0.139) | 4.003 (0.207) |
| Drug-8 | 6.061 (0.188) | 1.399 (0.179) | 4.662 (0.26) |
| Drug-9 | 6.093 (0.179) | 1.326 (0.18) | 4.767 (0.254) |


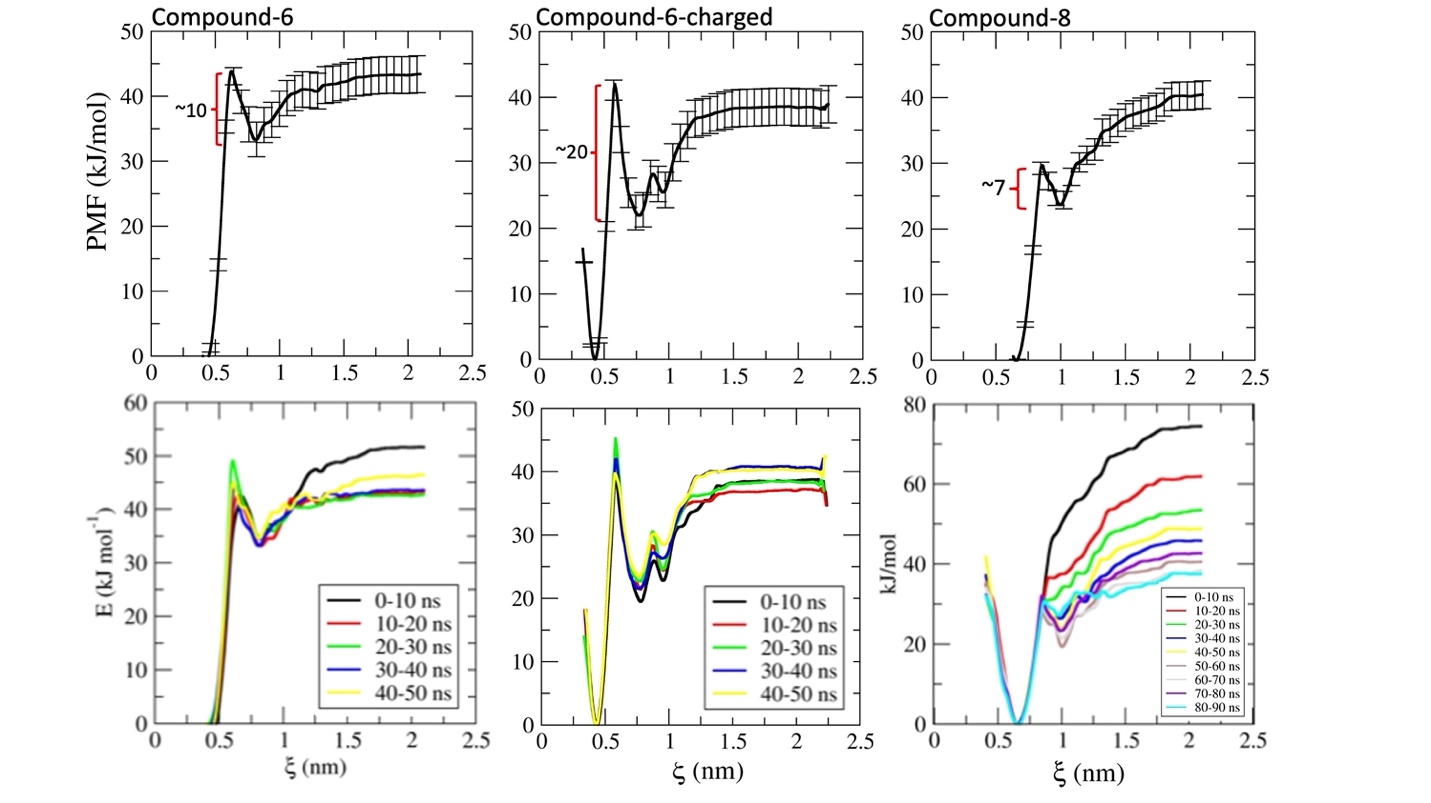


**Fig A**


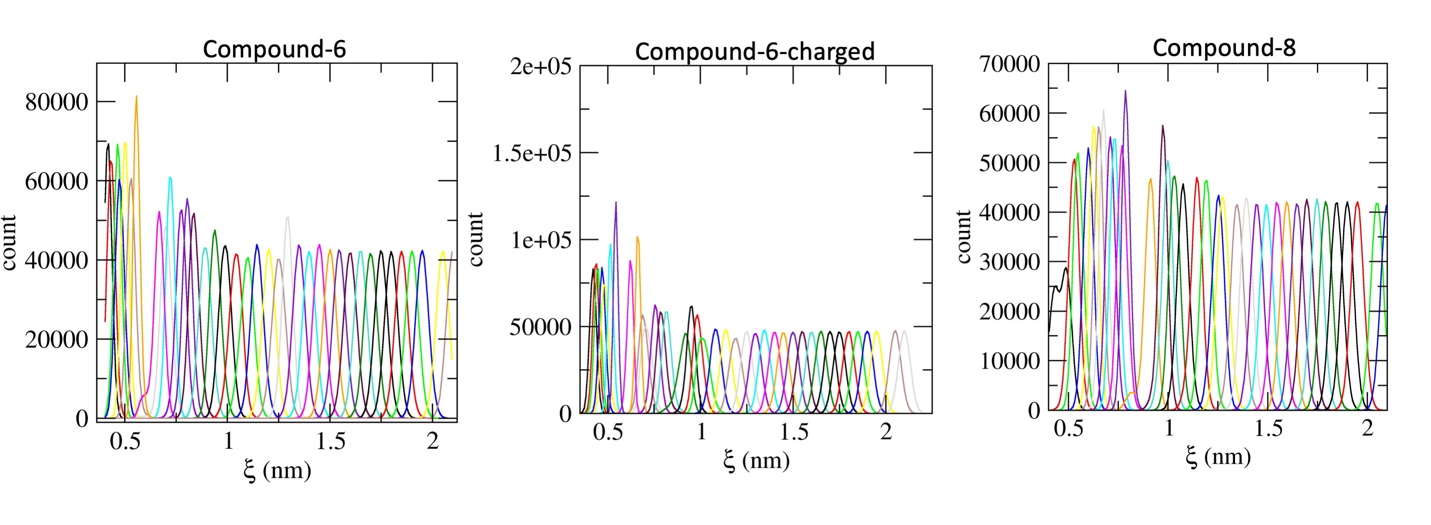


**Fig B**

**
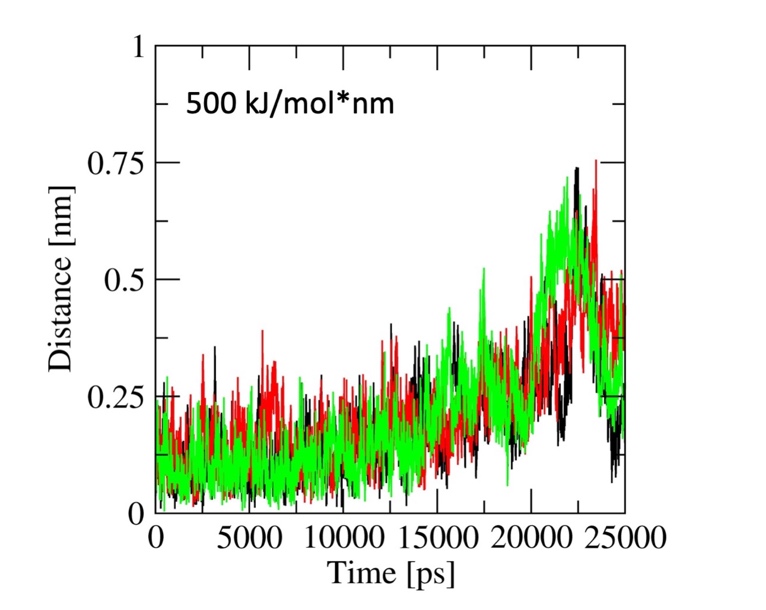
**

**Fig C**

**
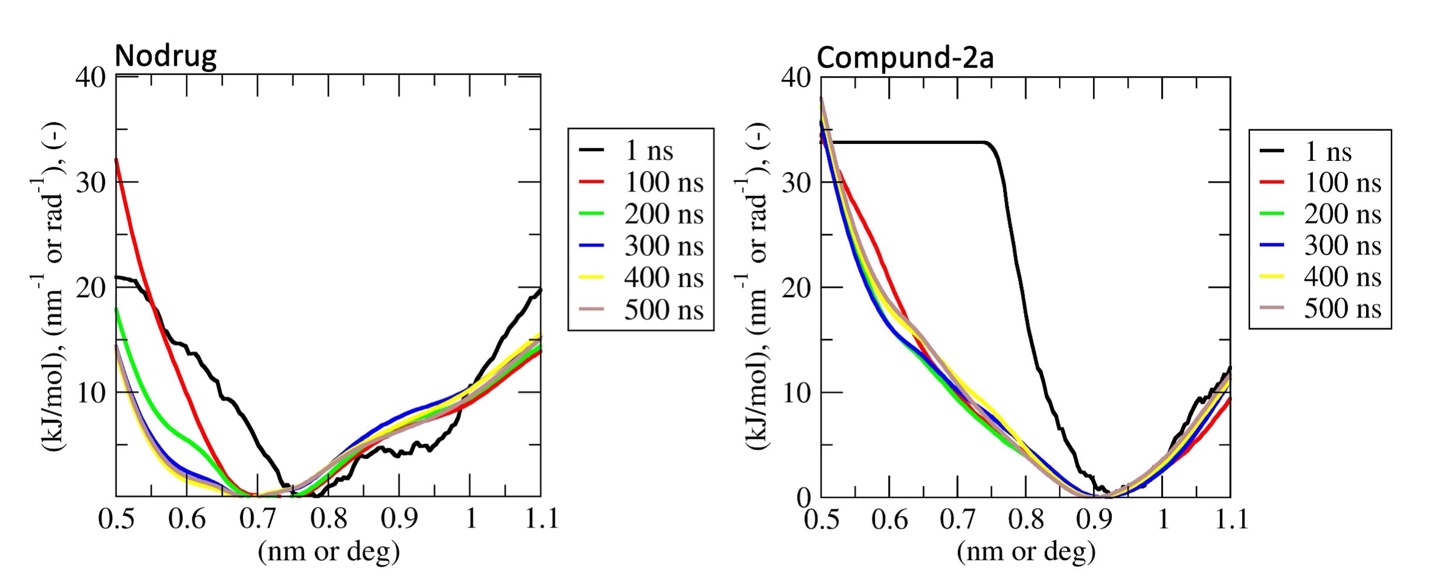
**

**Fig D**

**Umbrella sampling methods**

The LCAT together with bound drug 6 or 8 was orientated so that the longest axis of the drug molecules was parallel to the X-axis of the simulation box. In this way also the longest axis of the allosteric site was parallel to X-axis of the simulation box. Position restraints of 500 kJ/mol*nm for the backbone atoms of LCAT was introduced expect for the MBD. To generate the umbrella windows for sampling the drug molecules were pulled out from the allosteric site using pulling speed and force constant of 0.001 nm/ps and 5000 kJ/mol*nm^2^, respectively. The reference position was the center of mass of MBD and the center of mass of the drug molecule was used to pull molecules from the allosteric site. Molecules were pulled until the distance to the reference was ~2.1 nm. The pathway was then divided into umbrella windows with widths of 0.05 nm. Consequently. 34 umbrella windows were generated. Each umbrella window was sampled up to 50 and 90 ns in the case of compound 6 or 8, respectively. Thus, the corresponding total simulation sampling times were 1700 ns or 3060 ns. The force constant in each window was set to 2500 kJ/mol*nm. Additional windows with higher force constants (5000 kJ/mol*nm) were added into the region showing a free-energy barrier to ensure proper sampling in the barrier region. The converge of the corresponding PMFs took place in 10 or 50 ns (Fig A). The overlap of umbrella histograms is shown in Fig B. After these time points the PMF profiles were constructed utilizing the weighted histogram analysis method incorporated into the GROMACS simulation package through analysis tool gmx wham. The error estimation was carried out by utilizing the bootstrapping technique with a number of bootstraps set to 200.
